# A multi-omic approach reveals utility of CD45 expression in prognosis and novel target discovery

**DOI:** 10.3389/fgene.2022.928328

**Published:** 2022-08-17

**Authors:** Ni Ye, Jie Cai, Yulong Dong, Huiyao Chen, Zhiyuan Bo, Xiaogang Zhao, Mingyang Xia, Mei Han

**Affiliations:** ^1^ Department of General Practice, The First Affiliated Hospital of Soochow University, Suzhou, China; ^2^ Department of Thoracic Surgery, Shanghai Pulmonary Hospital, Tongji University School of Medicine, Shanghai, China; ^3^ The Third Department of Hepatic Surgery, Eastern Hepatobiliary Surgery Hospital, Second Military Medical University, Shanghai, China; ^4^ Molecular Medical Center, Children’s Hospital of Fudan University, Shanghai, China; ^5^ Department of Hepatobiliary Surgery, the First Affiliated Hospital of Wenzhou Medical University, Wenzhou, China; ^6^ Key Laboratory of Birth Defects, Children’s Hospital of Fudan University, Shanghai, China

**Keywords:** CD45, cancer immunotherapy, tumor microenvironment, biomarker, prognosis, pancancer

## Abstract

CD45, the leukocyte common antigen, is expressed on almost all cells of the immunological and hematological systems. CD45 expression is related to a variety of diseases, including leukemia and lymphoma. In this study, we analyzed the expression level of CD45 across cancers and evaluated the relationship between its expression and patient prognosis. We further integrated methylation data to explore the differences in CD45 across cancers from a multi-omics perspective. We also analyzed the relationship between CD45 expression and levels of immune cell infiltrates and immune modifiers. Our results revealed the distinct expression characteristics and prognostic value of CD45 across multiple tumors. In addition, we screened drug targets based on the immune index defined by CD45 expression and identified that GPR84 affected the proliferation of tumor cells and was associated with the inflammation caused by immunotherapy. In summary, our findings provide a comprehensive understanding of the role of CD45 in oncogenesis and its prognostic significance across cancers.

## Introduction

Cancer arises from the clonal expansion of abnormal cells with the accumulation of genomic abnormalities (Martincorena and Campbell, 2015). These cells can hijack homeostatic mechanisms to maintain their survival and proliferation (Hanahan and Weinberg, 2011). Tumor progression is mediated by many factors from the interior and exterior of tumor lesions, which constitute an intricate regulatory environment known as the tumor microenvironment (TME). The TME is a complex ecosystem that contains cancerous and noncancerous cells. This ecosystem plays an important role during tumor initiation and progression (Fridman et al., 2012). Cancer cells can escape immune supervision through cell–cell interactions. These escape phenomena are major obstacles to successful cancer therapy, especially during immunotherapy (Cao et al., 2020; Zeng et al., 2020).

Immunotherapy produces a significant antitumor effect mainly mediated by the immune cells inside the tumor microenvironment that specifically recognize and attack cancer cells (Yost et al., 2019). Immunotherapy has high specificity and few side effects and utilizes the mechanisms of the immune system (Schirrmacher, 2019). The immune cells in the TME can be divided into tumor-antagonizing immune cells and tumor-promoting immune cells (Lei et al., 2020). Tumor-antagonizing immune cells are mainly composed of effector T cells, natural killer (NK) cells, dendritic cells (DCs), M1-polarized macrophages and N1-polarized neutrophils. As effector T cells, CD8 (+) cytotoxic T cells (CTLs) have been considered the main subset of lymphocytes that kill cancer cells with major histocompatibility complex class I molecules (MHC-Is). Regulatory T cells (Tregs) and myeloid-derived suppressor cells (MDSCs) are the main components of tumor-promoting immune cells. Tregs are highly heterogeneous and clonally expanded in tumors and are closely related to the prognosis of patients (Zheng et al., 2017; Guo et al., 2018; Zhang et al., 2018). In addition, Tregs play a crucial role in maintaining immune homeostasis (Kim et al., 2007; Lahl et al., 2007).

CD45, also named protein tyrosine phosphatase receptor type C (PTPRC), is the leukocyte common antigen (Rheinlander et al., 2018). It is expressed on almost all cells of the immunological and hematological systems except for mature erythrocytes and platelets (Barford et al., 1994). Changes in CD45 expression are associated with a variety of diseases, including leukemia and lymphoma (Ozdemirli et al., 1996; Ratei et al., 1998; Rheinlander et al., 2018). Different types of leukemia and stem cell transplantation have been treated by blocking the function of CD45 with specific antibodies or inhibitors (Ostergaard and Trowbridge, 1991; Autero et al., 1994; Hermiston et al., 2003). Human CD45 mRNA has six isoforms, and the alternative expression of different types of isoforms plays an important role during HSC proliferation and differentiation (Dornan et al., 2002; Hermiston et al., 2003). CD45 is also an essential regulator of immune function that influences lymphocyte survival, cytokine responses, and TCR signaling (Tchilian and Beverley, 2006; Saunders and Johnson, 2010). For example, the interaction between T cells and macrophages is regulated by CD45 via the ligand–receptor pathway and attenuates T-cell proliferation (Schuette et al., 2016). The above studies indicate that CD45 is an important regulator in the immune system. However, there is no systematic pan-cancer research on CD45.

In this study, we analyzed the expression level of CD45 across cancers and evaluated the relationship between its expression and patient prognosis. We further integrated methylation data to explore the variations in CD45 across cancers from a multi-omics perspective. We also analyzed the relationship between CD45 expression and levels of immune cell infiltrates and immune modifiers. Finally, we screened drug targets based on the immune index defined by CD45 expression and identified that the immune-related gene GPR84 affected the proliferation of cancer cells and was associated with the inflammation brought during immunotherapy. In general, our study reveals the landscape of CD45 expression across different cancers and demonstrates its utility in prognosis and target discovery.

## Materials and methods

### Data collection

RNA-seq data of 10,730 tumor tissues from The Cancer Genome Atlas (TCGA) and Therapeutically Applicable Research to Generate Effective Treatments (TARGET) and 4,976 normal tissues from GTEx were downloaded from the UCSC Xena website (https://xenabrowser.net/datapages/). DNA methylation data were obtained from the cBioPortal database (https://www.cbiop ortal. org/). The clinical information for cancer patients could also be downloaded from the UCSC Xena website. The abbreviation and full name of all cancers in TCGA are presented in Supplementary Table S1.

### Analysis of CD45 expression patterns in tumor and normal tissues

CD45 mRNA expression (TPM, transcripts per million) from “TcgaTargetGtex_rsem_gene_tpm dataset” was quantified. The expression data from this dataset was recalculated and normalized. The median expression level of CD45 was determined to compare the expression changes between tumor and normal tissues. The adjusted *p* value (adj. P) < 0.05 was considered statistically significant.

### Protein level analysis of CD45

The protein expression data of CD45 was assessed based on immunohistochemistry using tissue microarrays in human tumor and normal tissues were obtained from The Human Protein Atlas (HPA: https://www.proteinatlas.org/). The histogram of CD45 expression at the protein level was constructed by GraphPad 8.3.0.

### Analysis of CD45 expression patterns in cell lines

Transcriptional data of CD45 expression in different cell types were downloaded from The Human Protein Atlas (HPA: https://www.proteinatlas.org/). Normalized expression data of CD45 levels in cancer cells and normal cells and transcripts per million data of CD45 in blood cells (HPA, Monaco and Schmiedel project) were analyzed. The histogram of CD45 expression at the transcriptional level was plotted by GraphPad 8.3.0.

### Pan-cancer prognostic analysis of CD45

Kaplan-Meier analysis was performed to evaluate the relationship between CD45 expression (high and low expression group based on median value of CD45 expression) and the overall survival (OS) of patients from the TCGA cohort. Univariate Cox regression analysis was performed using the R package “survival” to assess the significance of CD45 (expression value of CD45 as a continuous variable) in predicting OS, disease-specific survival (DSS), disease-free interval (DFI), and progression-free interval (PFI) across cancers.

### Methylation analysis of CD45

The profile of CD45 methylation variations was interpreted based on the DNA methylation data of CD45 downloaded from the UCSC Xena website. Information on the methylation probe was obtained by the R package “IlluminaHumanMethylation450kanno.ilmn12. hg19”.

### Gene set enrichment analyses

Each type of tumor in TCGA was divided into high and low expression groups according to the median expression level of CD45 in TCGA. The differentially expressed genes were obtained by the “limma” package. Gene set enrichment analysis (GSEA) and Kyoto Encyclopedia of Genes and Genomes (KEGG) analysis were performed based on differentially expressed gene sets. Genes with the adjusted *p* value (adj. P) < 0.05 were selected for GSEA.

### Immune cell infiltration analysis

The immune cell infiltration score was calculated by the xCell method (Aran et al., 2017). Samples of each tumor in TCGA were divided into two groups based on the median CD45 expression to compare the level of immune cell infiltration. The correlation between CD45 expression and CD8 (+) T cells, CD4 (+) T cells and T (regs) was explored by Tumor Immune Estimation Resource 2.0 (TIMER2.0; http://timer.comp-genomics.org). Correlation analysis between expression levels of CD45 and levels of chemokines, immunoinhibitory factors, immunostimulatory factors, MHC and receptors in each cancer was performed by the R package “psych”.

### Analysis of the relationship between CD45 expression and TMB, MSI, MMR and TNB

The SangerBox website (http://sangerbox.com/Tool) was used to explore the relationship between CD45 expression and tumor mutational burden (TMB), microsatellite instability (MSI), mismatch repair (MMR) and tumor neoantigen burden (TNB).

### Cell viability assay

The viability of cancer cell lines (A549, U87-MG and 293T) treated with GPR84 antagonist (GPR84 antagonist 1 (Jenkins et al., 2021), MedChemExpress) or vehicle (DMSO, Sigma) was calculated by Cell Counting Kit-8 (Japan Dojindo) according to the manufacturer’s protocol. Briefly, A549 (1.5 × 10^3^/well), 293T (1.5 × 10^3^/well) and U87-MG cells (2 × 10^3^/well) were seeded into 96-well cell culture plates and treated with GPR84 antagonist (GPR84 antagonist 1 (Jenkins et al., 2021), MedChemExpress) or vehicle (DMSO, Sigma) at 37 °C in 5% CO_2_ humidified atmosphere. At each detection time point, CCK-8 reagent was added to the medium and incubated for 1–2 h at 37 °C in 5% CO_2_ humidified atmosphere. The OD value of each well in 96-well cell culture plates was obtained by multifunctional microporous plate detector (SynergyLX, BioTek).

### Plate clone formation assay

A549 (3 × 10^3^/well), 293T (3 × 10^3^/well) and U87-MG (3 × 10^3^/well) cells were seeded into 6-well cell culture plates. GPR84 antagonist (GPR84 antagonist 1, MedChemExpress) or vehicle (DMSO, Sigma) was added to DMEM containing 10% FBS after cell adherence. Two weeks later, the cancer cell lines (A549, U87-MG and 293T) were stained with crystal violet. Images of colonies in each well were obtained under an optical camera.

### Statistical analysis

All experimental data are represented as the mean ± standard deviation (SD). Statistical analysis was performed by Student’s t test or analysis of variance (ANOVA). The differential expression level of CD45 between tumor tissues and normal tissues was analyzed by the Mann–Whitney *U* test. Kaplan–Meier survival analysis was used to estimate the survival time of tumor patients based on high or low levels of CD45 expression in TCGA. For prognosis analysis, the HR and *p* value were calculated using univariate Cox regression analysis to investigate the relationship between CD45 expression and prognostic indicators (OS, DSS, DFI, PFI). A *p* value <0.05 was considered statistically significant.

## Results

### Expression profile of CD45 in tumor and normal tissues

**FIGURE 1:**
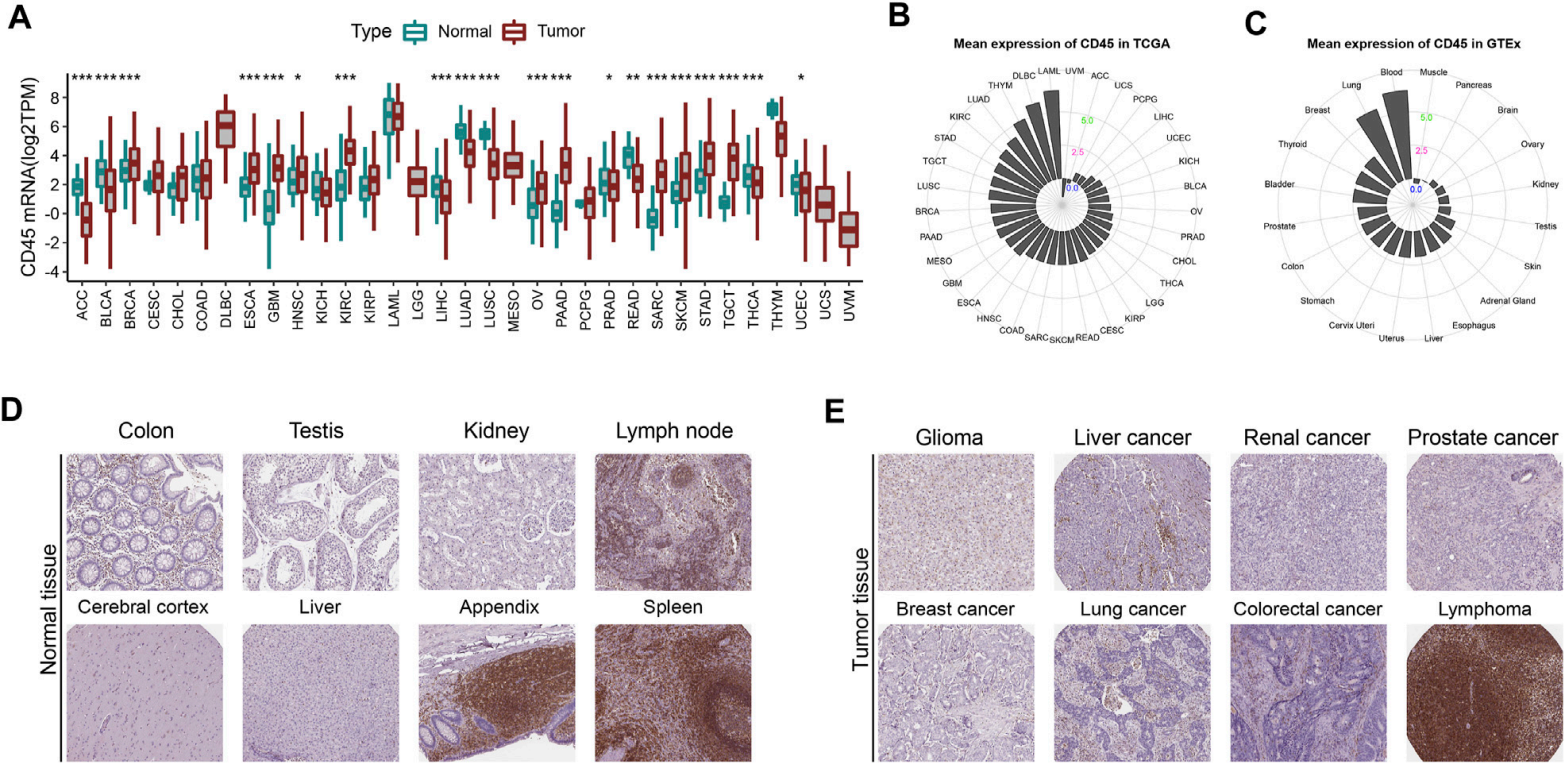
CD45 expression profiles in human normal and tumor tissues. **(A)** Pan-cancer analysis of CD45 expression in tumor tissues from TCGA and normal tissues from GTEx. **(B)** CD45 expression in human tumor tissues from TCGA. The location of the dot represents the mean expression value of CD45 mRNA. **(C)** CD45 expression in human normal tissues from GTEx. The location of the dot represents the mean expression value of CD45 mRNA. The protein expression level of CD45 in human normal tissues **(D)** and tumor tissues **(E)** is displayed in the immunohistochemical images. *p < 0.05; **p < 0.01 and ***p < 0.001.

To comprehensively depict the CD45 expression profile in tumor and normal tissues, we first analyzed the mRNA expression level of CD45 in tumor tissues from TCGA and normal tissues from GTEx. The results showed that the expression level of CD45 was significantly higher in multiple tumor tissues than in normal tissues, such as BRCA, ESCA, GBM, HNSC, KIRC, PAAD and SARC (Figure 1A). We also observed lower expression levels of CD45 in ACC, BLCA, LIHC, LUAD, LUSC and READ than in normal tissues (Figure 1A). For tumor and normal tissues, we found that CD45 was highly expressed in LAML and blood, respectively (Figures 1B,C). Interestingly, there was no significant difference in CD45 expression between LAML and blood, which may be due to the high abundance of CD45 (+) cells under both physiological and pathological conditions (Figure 1A). CD45 belongs to the cell surface transmembrane protein family, which can transmit cell signals and affect cell proliferation and differentiation (Al Barashdi et al., 2021). Therefore, we analyzed the protein expression level of CD45 in normal and tumor tissues through the HPA database. We downloaded the expression data of CD45 protein detected by immunohistochemical staining. After analysis, we discovered that CD45 was highly expressed only in some normal tissues but not in other normal tissues (Figure 1D; Supplementary Figure S1A). Similarly, CD45 was highly expressed only in hematological tumors and not detected in other types of tumors (Figure 1E; Supplementary Figure S1B). These results showed that the transcriptional level of CD45 varied in different tumors and its protein expression was concentrated in specific tissues.

### CD45 is highly expressed in immune cells

**FIGURE 2:**
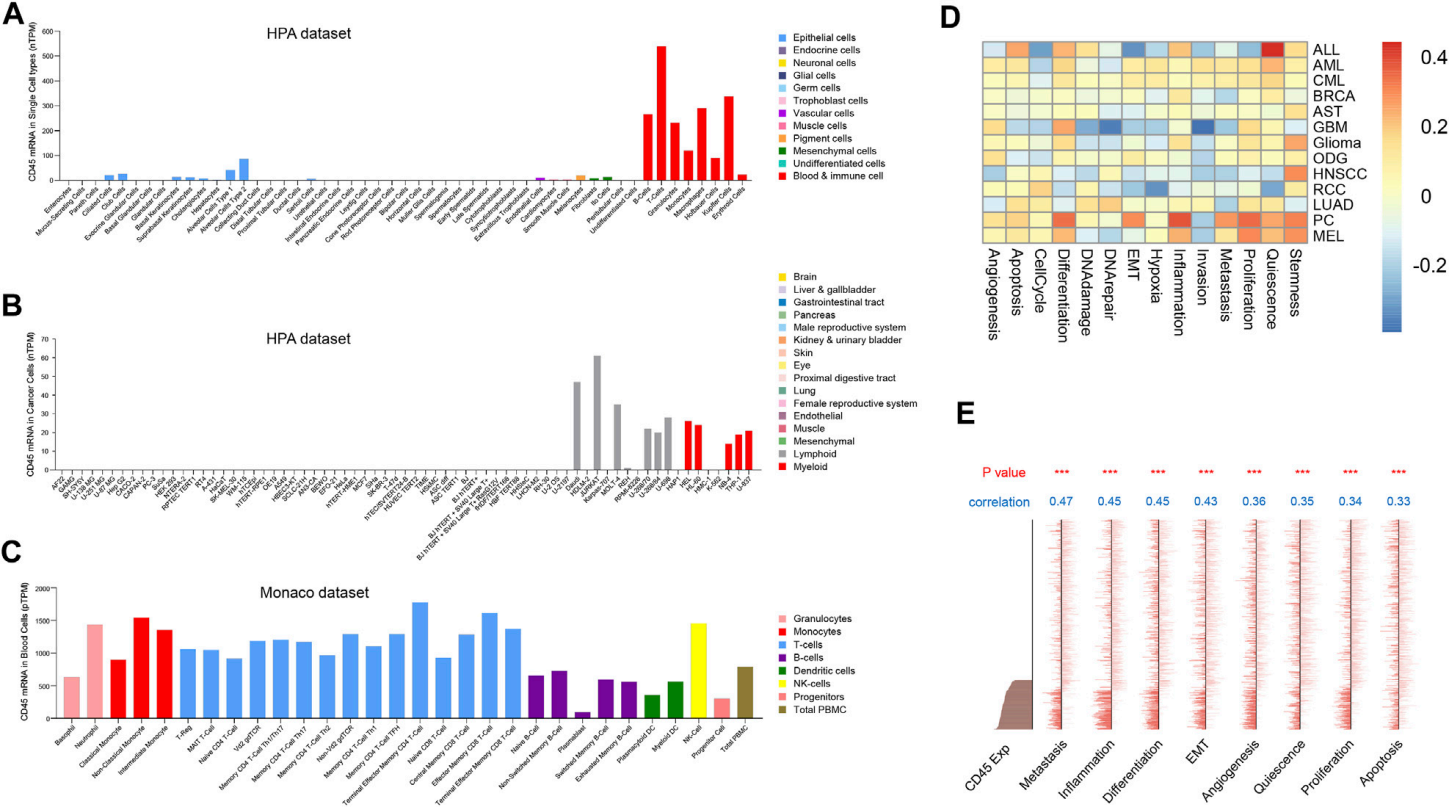
Analysis of CD45 expression at the cellular level. Analysis of CD45 expression in normal cells **(A)**, cancer cells **(B)** and immune cells **(C)** based on transcriptome data. **(D)** Analysis of correlation between CD45 expression and the occurrence of multiple cellular processes in different cancers based on single-cell sequencing data in CancerSEA. **(E)** Analysis of correlation between CD45 expression and the occurrence of multiple cellular processes in AML (EXP0047) based on single-cell sequencing data in CancerSEA. *p < 0.05; **p < 0.01 and ***p < 0.001.

Tumor and normal tissues contain different cell types and tumor tissues have higher cellular heterogeneity (Meacham and Morrison, 2013). The difference in CD45 expression between tumor and normal tissues further prompted us to explore CD45 expression at the cellular level in normal and cancer tissues. We first analyzed the transcriptome data of normal cell from the human protein database and found that CD45 was mainly expressed in immune cells, which was consistent with results of previous study. CD45 was also detected in endothelial cells, mesenchymal cells and pigment cells with low expression levels (Figure 2A). In addition, the expression of CD45 in some cancer cell types, such as Hela, HepG2 and MCF7 cells, was not observed (Figure 2B). The expression level of CD45 also varied in different immune cells. In general, the expression level of CD45 in monocytes and T cells was higher than that in B cells and DC cells in blood (Figure 2C). Furthermore, we discovered that the expression level of CD45 was the highest in nonclassical monocytes by analyzing multiple datasets, including the HPA, Monaco and Schmiedel datasets (Figure 2C, Supplementary Figures S2A, B). Moreover, the expression level of CD45 was positively correlated with the occurrence of multiple cellular processes in some tumors based on single-cell sequencing data, including proliferation, differentiation, inflammation, stemness and angiogenesis (Figure 2D). In AML, the expression level of CD45 was significantly related to metastasis, inflammation, differentiation and EMT (Figure 2E). These results suggested that CD45 was not only highly expressed in immune cells but also associated with multiple cellular processes.

### The pan-cancer landscape of CD45 methylation

DNA methylation is an epigenetic mechanism that mediates regulation of gene expression under physiological and pathological conditions (Jones, 2012). With the development of sequencing technology, we can better detect and analyze changes in DNA methylation. The methylation data (Illumina Human Methylation 450k) from the TCGA database was downloaded to investigate whether CD45 expression across different cancers could be correlated with DNA methylation by the R package “IlluminaHumanMethylation450 kanno. ilmn12. hg19”. The results showed high negative correlation between the CD45 methylation level and its expression level in a variety of tumors, which indicated high methylation of CD45 and low CD45 expression in these tumors (Supplementary Figure S3A; Figure 1B). We also analyzed the correlation between CD45 and methyltransferase genes at the transcriptional level. The results showed that there was high correlation between CD45 and methyltransferase genes in some tumors, but there was no significant correlation in other tumors (Supplementary Figure S3B).

Previous results showed that expression level of CD45 was higher in TGCT and PAAD than that in UCS and PCPG (Figure 1B). We also found that the correlation between the CD45 methylation level and its expression was low in TGCT and PAAD and high in UCS and PCPG (Supplementary Figure S3A). These results implied that DNA methylation played an important role in CD45 expression among some tumors.

### Correlation between the expression Level of CD45 and clinicopathological features of tumor patients

To investigate the relationship between the expression level of CD45 and clinicopathological features, we first compared the expression level of CD45 between male and female tumor patients from TCGA. The results revealed that the expression level of CD45 in male tumor patients was higher than that in female tumor patients in MESO, PCPG and SARC. In some tumors, the expression level of CD45 in males was lower than that in females, including BLCA, BRCA, HNSC and LUSC. In addition, there was no significant difference in CD45 expression between males and females in some tumors (Supplementary Figure S4A).

With increasing age, more gene mutations accumulate in the body, and this phenomenon may affect gene expression, especially in tumor patients (Martincorena and Campbell, 2015; Rodriguez et al., 2017). Therefore, we divided the patients into two groups according to their age (≥65 vs. < 65) and compared the expression level of CD45 between the two groups. We found that CD45 expression was higher in older patients (age ≥65) in ESCA, LAML and LUAD (Supplementary Figure S4B).

Tumors can be divided into different stages based on clinical diagnosis. We screened tumor patients with tumor stage information in TCGA and compared CD45 expression across different stages. The results showed that the expression level of CD45 decreased with tumor stage from grade I to grade IV in ACC and TGCT. Moreover, there was no significant difference from grade I to grade IV in most other tumors (Supplementary Figure S4C).

### The expression level of CD45 is related to prognosis in multiple tumors

**FIGURE 3:**
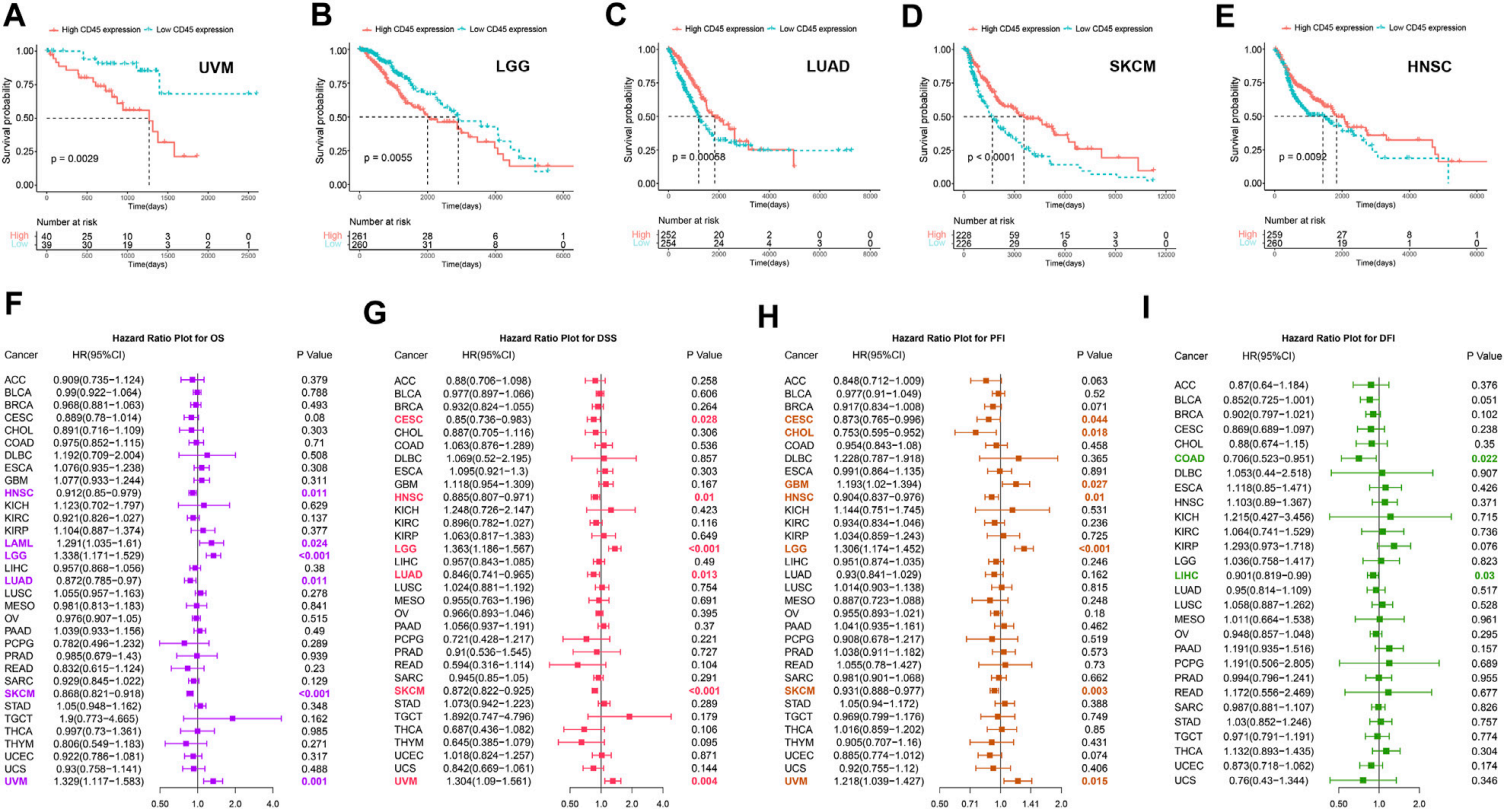
Analysis of the prognostic value of CD45 across different cancers. Survival curves plotted by the Kaplan-Meier method in UVM **(A)**, LGG **(B)**, LUAD **(C)**, SKCM **(D)** and HNSC **(E)** between the high and low CD45 expression groups. The value of CD45 expression and OS **(F)**, DSS **(G)**, PFI **(H)** and DFI **(I)** prognostic factors assessed by univariate Cox regression analysis.

To clarify the prognostic impact of CD45, we analyzed the relationship between patient prognosis and CD45 expression across cancers by the Kaplan-Meier method. The results showed that patients with high CD45 expression had shorter survival time in UVM and LGG (Figures 3A,B). On the other hand, patients with low CD45 expression had shorter survival time in LUAD, SKCM and HNSC (Figures 3C–E). This phenomenon might be due to the difference between tumors, including different pathogenic mechanism, different location of tumor and tumor heterogeneity. To further examine the prognostic potential of CD45, we used univariate Cox regression analysis to determine the prognostic relationship between CD45 expression and prognostic indicators (OS, DSS, PFI, DFI). The OS analysis results revealed that CD45 served as a hazard factor for patients with LAML, LGG or UVM (Figure 3F). The DSS analysis results showed that CD45 acted as a protective factor for patients with CESC, HNSC, LUAD or SKCM (Figure 3G; Supplementary Figures S5A–E). The PFI analysis results showed that CD45 was an unfavorable factor for patients with GBM, LGG or UVM (Figure 3H, Supplementary Figures S5F–J). The DFI analysis results indicated that CD45 worked as a protective factor for patients with COAD or LIHC (Figure 3I; Supplementary Figures S5K–O). Taken together, the results showed that the expression level of CD45 was associated with prognosis in multiple cancers.

### Pan-cancer analysis of the potential mechanism of CD45 function in the tumor microenvironment

Previous studies have shown that CD45 affects cell differentiation and proliferation in hematopoietic stem cells (HSCs) (Dornan et al., 2002; Hermiston et al., 2003). To further evaluate the potential molecular mechanisms of CD45, we first divided each tumor into high and low expression groups according to the median expression level of CD45 and obtained the differentially expressed genes in each tumor by bioinformatics methods. Then, we performed GSEA and KEGG analysis to discover potential molecular mechanisms based on the differentially expressed gene sets. We found that CD45 was significantly associated with immune-related pathways in multiple tumors, such as BRCA, HNSC, COAD, GBM, ESCA, and PAAD (Supplementary Figures 6A, B). The uncontrolled proliferation of tumor cells is the main feature of tumor progression. Therefore, we detected the correlation between the expression levels of CD45 and MKI67, which is a marker of cell proliferation. The results showed that there was high correlation between CD45 and MKI67 expression levels in DLBC, HNSC, KIRC, LAML, LIHC, PRAD, THCA, THYM and UVM (Supplementary Figure S6C).

### High CD45 expression correlates with immune infiltration across cancers

**FIGURE 4:**
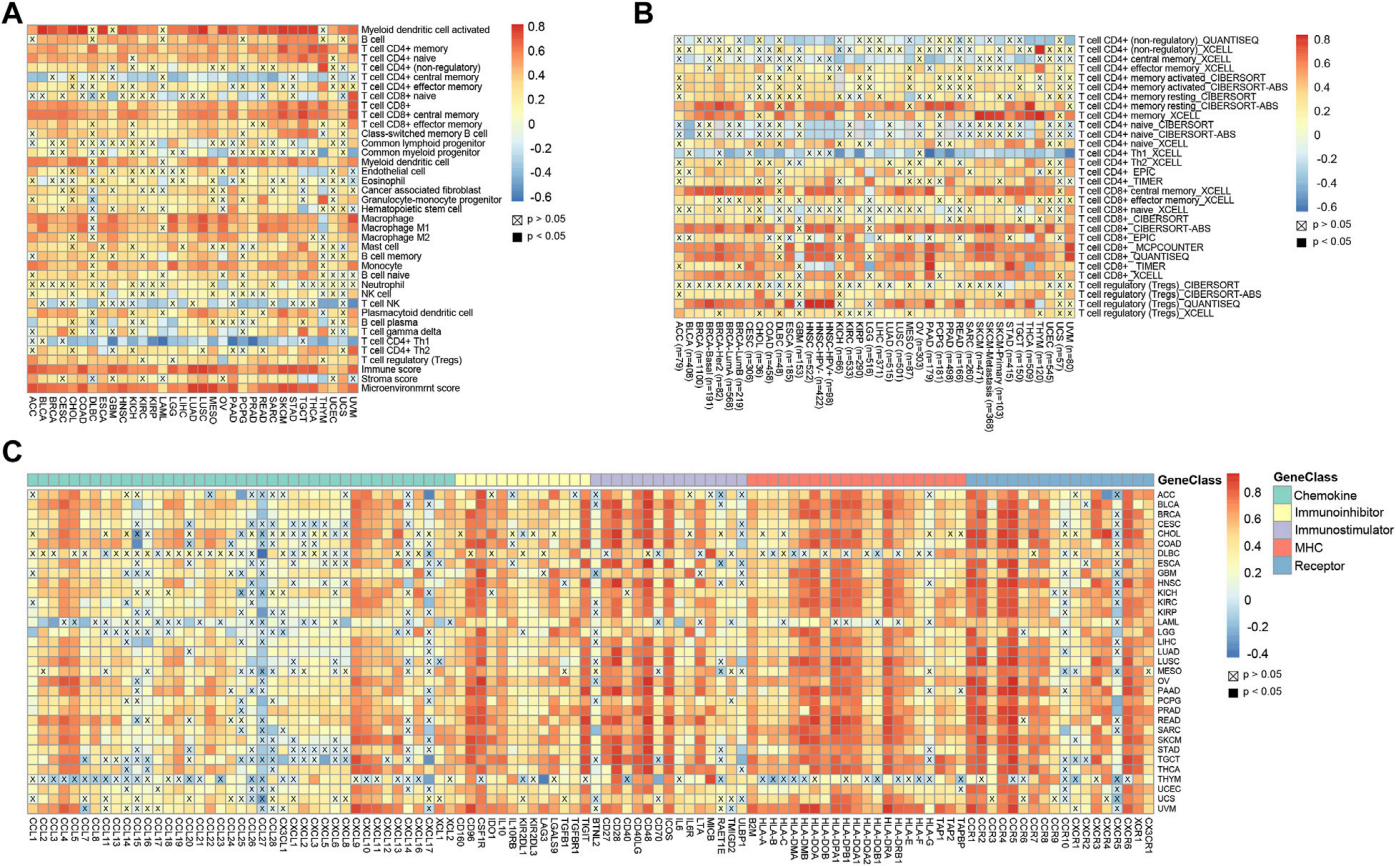
High CD45 expression correlates with immune infiltration across different cancers. **(A)** Analysis of correlation between CD45 expression and immune infiltration, stromal score, immune score, and Microenvironment score across cancers. **(B)** CD45 expression significantly correlated with the infiltration levels of various immune cells in the TIMER database. **(C)** Analysis of correlation between the expression level of CD45 and levels of immunomodulators across cancers.

The tumor immune microenvironment affects tumor progression in different ways (Hinshaw and Shevde, 2019). Therefore, we compared the correlation between CD45 expression and the presence of various immune infiltrates calculated by the xCell method across cancers. Overall, the pan-cancer expression level of CD45 was positively correlated with the presence of multiple immune infiltrates, such as CD4 (+) memory T cells, CD8 (+) T cells, myeloid dendritic cells and macrophages. In contrast, negative correlation was found between CD45 expression and the presence of CD4 (+) central memory T cells, NK cells and Th1 CD4 (+) T cells. In addition, significant positive correlation was found between CD45 expression and the presence of Th2 CD4 (+) T cells. These results also further confirmed the differential effects of Th1 CD4 (+) T cells and Th2 CD4 (+) T cells in maintaining homeostasis (Figure 4A). In addition, TIMER2.0 was used to depict the correlation landscape between CD45 expression and the presence of mainly immune cells, including CD8 (+) T cells, CD4 (+) T cells and Tregs. In general, the expression level of CD45 was positively correlated with the presence of CD8 (+) T cells and T (regs) and negatively correlated with the presence of CD4 (+) T cells in a variety of tumors (Figure 4B).

T-cell exhaustion often occurs in patients with chronic infection and cancer. The main reason for this phenomenon is that T cells cannot effectively develop and differentiate due to the continuous stimulation of antigens. Tumor patients have a lot of T cells in their bodies, but most of the T cells are exhausted (Wherry, 2011; Wherry and Kurachi, 2015). To determine the relationship between CD45 and exhausted T cells, we performed correlation analysis of CD45 expression and the levels of immunomodulators, including chemokines, immune inhibitors, immune stimulators, MHCs and partial receptors. The results revealed that CD45 was positively correlated with the expression levels of genes characteristic of exhausted T cells across cancers, such as CXCL9, IL10, CD28, IDO1 and CCR4 (Figure 4C).

### Correlation between CD45 Pan-cancer expression and TMB, TNB, MSI and MMRs

Immunotherapy is new method of tumor treatment developed after surgery, radiotherapy and chemotherapy (Kennedy and Salama, 2020; Nam et al., 2020). TMB, TNB, MSI and MMR have good predictive effect in tumor immunotherapy (Samstein et al., 2019; Schrock et al., 2019; Lizardo et al., 2020). We first analyzed the correlation between the expression level of CD45 and TMB. The results showed that CD45 had high positive correlation with TMB in COAD, UCEC, OV and SARC and negative correlation in THYM, UVM and PAAD (Supplementary Figure S7A). Tumors with high TMB tend to produce tumor neoantigens. Tumors with more tumor neoantigens have higher immunogenicity and are more sensitive to immunotherapy. Therefore, we detected the relationship between the expression level of CD45 and levels of tumor neoantigens and found that CD45 had a positive correlation with levels of tumor neoantigens in UCEC and OV and negative correlation in READ (Supplementary Figure S7B). This result was also consistent with the correlation observed between the expression level of CD45 and TMB in these tumors. In addition, we also found that the expression level of CD45 was higher in tumors with high MSI, such as COAD, READ and LAML (Supplementary Figure S7C). MMR is another marker of tumor immunotherapy. Therefore, we analyzed the relationship between CD45 expression levels and expression levels of MMR-related genes and found high correlation between levels of CD45 and MMR-related genes in multiple tumors (Supplementary Figure S7D).

### Drug target screening based on the immune index defined by CD45 expression

Surgical resection, radiotherapy and chemotherapy are traditional treatment methods for cancer. Targeted therapy and immunotherapy based on specific gene targets have emerged as treatment methods in recent years. However, some patients with cancer still have a poor prognosis after receiving these treatments (Lim et al., 2005). Drug target screening based on the immune index is helpful for the combination of targeted therapy and immunotherapy, but there is no relevant evidence of this approach at present.

**FIGURE 5:**
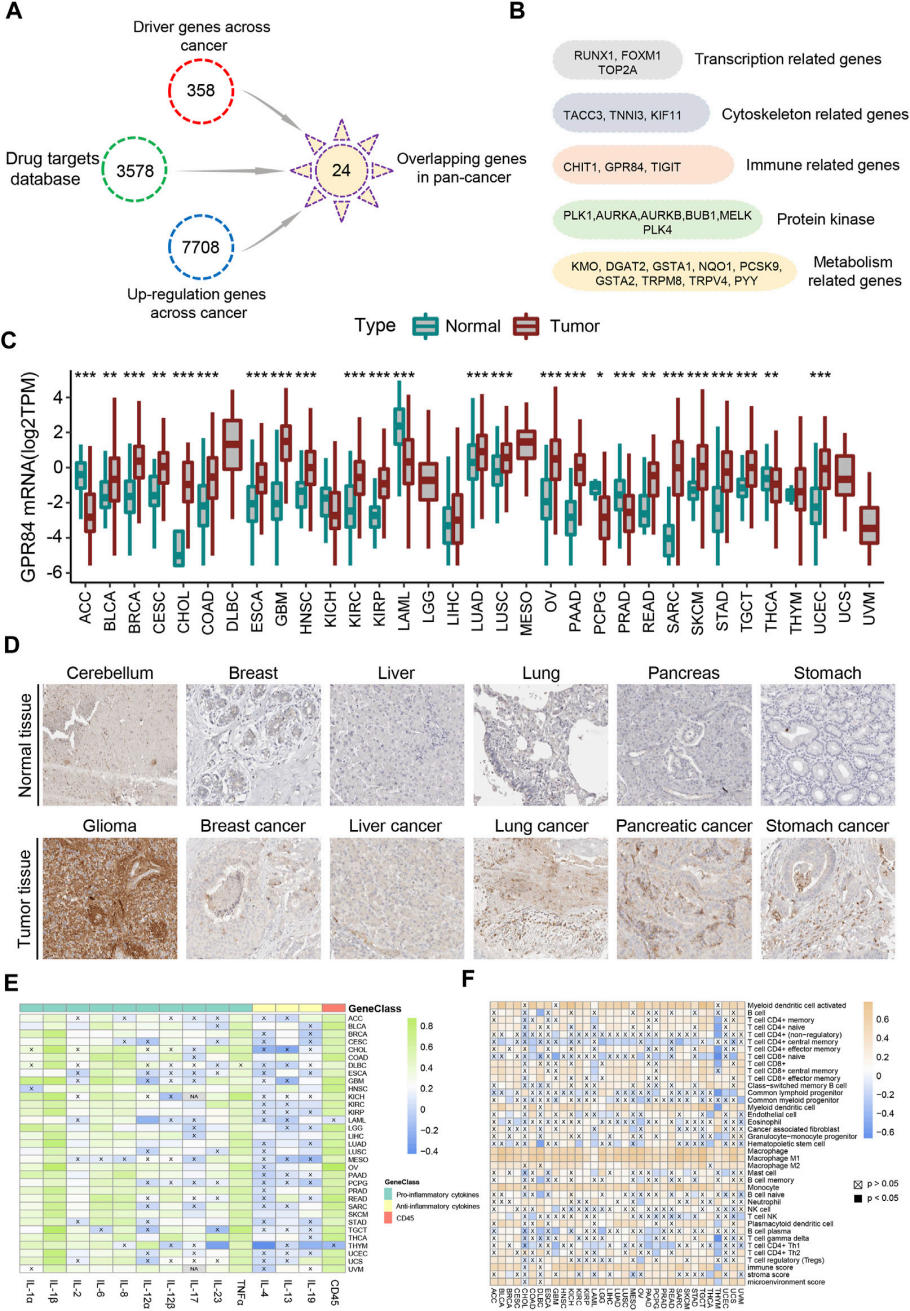
Screening of drug targets based on the immune index defined by CD45 expression. **(A)** Screening flowchart of potential drug targets based on the immune index between the normal tissue and the high or low immune index groups across cancers. **(B)** 24 common potential drug targets across different cancers divided into five categories: transcription-related genes, cytoskeleton-related genes, immune-related genes, protein kinase and metabolism-related genes. The mRNA **(C)** and protein **(D)** levels of GPR84 in normal tissue and tumor tissue across cancers from the Human Protein Atlas. Analysis of correlation between the expression level of GPR84 and levels of inflammatory factors **(E)** and immune cells **(F)** across cancers. *p < 0.05; **p < 0.01 and ***p < 0.001.

CD45 is the surface marker of immune cells, and its expression level is directly proportional to the number of immune cells in tumor tissue (Hermiston et al., 2003). Therefore, we divided each cancer into high immune index group and low immune index group based on the expression level of CD45 in TCGA. Our study focuses on screening drug targets based on the immune index across different cancers by multiple bioinformatics methods previously reported (Xia et al., 2021). Briefly, the upregulated genes between normal tissues and high or low immune index groups were screened by the pan-cancer R package “limma”. Then, we used the Netbid algorithm to screen for ‘hidden’ driver genes in normal tissues and high or low immune index groups across different cancers (Gocho et al., 2021). Next, we integrated the drug targets in the therapeutic target database (TTD) and identified 24 common potential drug targets across different cancers (Figure 5A). After gene function annotation, these genes can be divided into five categories: protein kinase, metabolism-related genes, gene transcription-related genes, immune-related genes and cytoskeleton-related genes (Figure 5B).

### GPR84 participates in the inflammatory response and affects tumor cell proliferation

Immunotherapy has brought benefits to the majority of tumor patients (Yang, 2015). However, some patients with cancer often suffer from inflammation for a long time after immunotherapy in clinical treatment, which may be a side effect of immunotherapy (Crusz and Balkwill, 2015; Dougan et al., 2021). Effectively overcoming inflammation after immunotherapy will help to improve the therapeutic effect in tumor patients (Nakamura and Smyth, 2017; Wattenberg and Beatty, 2020). Surprisingly, the immune-related gene GPR84, which is the one gene belonging to the G protein-coupled receptor family among the 24 potential drug targets, mediates inflammation and the innate immune response (Recio et al., 2018; Marsango et al., 2020; Zhang et al., 2021). Therefore, we believe that GPR84 plays an important role in the inflammatory response after immunotherapy. We first analyzed the expression level of GPR84 across cancers and found that the expression level of GPR84 was higher in glioma, breast cancer, liver cancer, lung cancer, pancreatic cancer and stomach cancer than in normal tissues (Figures 5C,D). Inflammatory factors, including proinflammatory (IL-1α, IL-1β, IL-2, IL-6, IL-8, IL-12α, IL-12β, IL-17, IL-23, TNFα) and anti-inflammatory factors (IL-4, IL-13, IL-19), are the main effectors in the inflammatory response (Medzhitov, 2010). Therefore, we detected the correlation between the expression level of GPR84 and inflammatory factors. The results showed that there was high correlation between expression level of GPR84 and levels of proinflammatory factors in several tumors. High correlation between expression level of GPR84 and CD45 was also observed across cancers (Figure 5E). In addition, we found that there was high correlation between the expression level of GPR84 and macrophage and myeloid dendritic cells in multiple cancers (Figure 5F). The above results suggest that GPR84 is involved in the inflammatory response.

**FIGURE 6:**
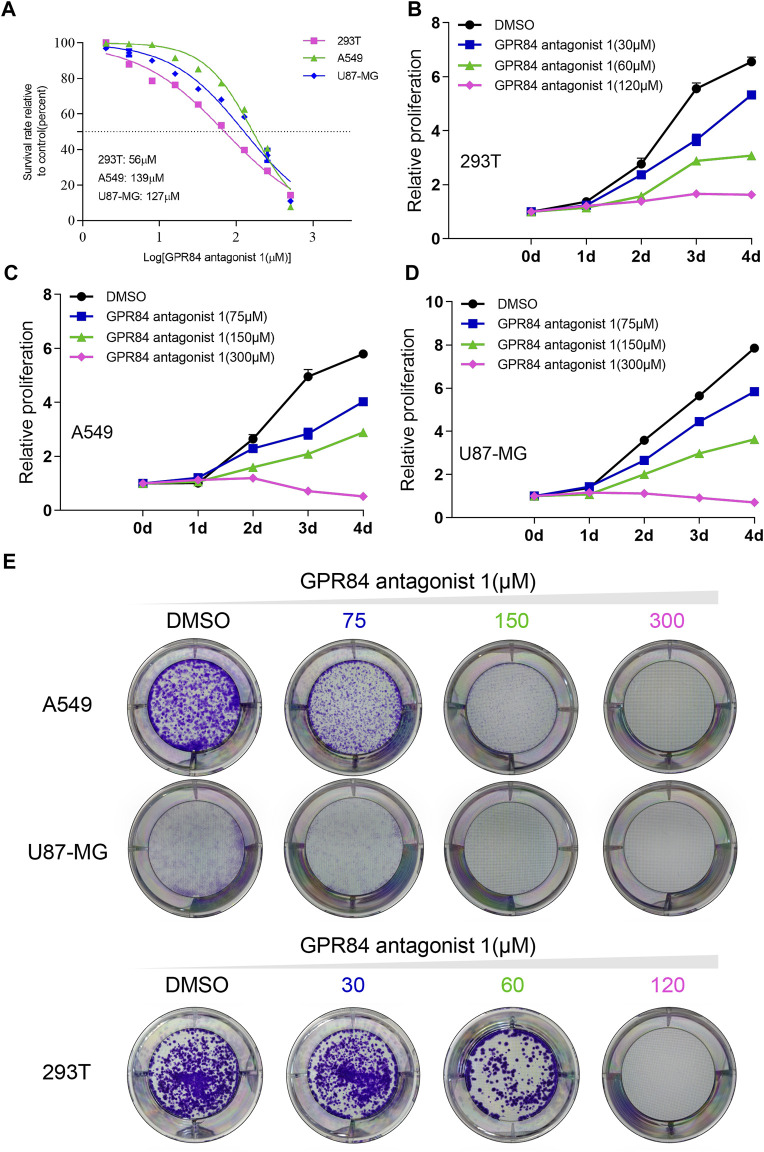
Blocking GPR84 reduced the proliferation of cancer cells. **(A)** IC50 curve of GPR84 antagonist one in A549, U87-MG and 293T cells. Cell proliferation of 293T **(B)**, A549 **(C)** and U87-MG **(D)** cells treated with GPR84 antagonist one or DMSO was tested by a cell viability assay. **(E)** The colony formation ability of A549, U87-MG and 293T cells treated with GPR84 antagonist one or DMSO was detected by plate clone formation assays.

Previous results have shown that GPR84 can be used as a potential drug target (Figure 5A). We found that expression level of GPR84 was higher in some tumor tissues than in normal tissues, such as glioma, breast cancer, liver cancer, kidney cancer, lung cancer, pancreatic cancer and stomach cancer. (Figures 5C,D). Therefore, we used an antagonist of GPR84 and selected A549, 293T and U87-MG cells as models to evaluate the feasibility of GPR84 as a potential drug target. We first obtained the IC50 value of the GPR84 antagonist in the 3 cell lines and determined three drug concentrations based on the IC50 value (Figure 6A). CCK8 assays and plate clone formation assays were used to evaluate the effect of GPR84 antagonists on cell proliferation. The results showed that the proliferation and clone formation ability of tumor cells decreased significantly after neutralizing the function of GPR84. The antagonistic effect became more obvious with increasing drug concentration (Figures 6B–E). The above results show that GPR84 is not only involved in the inflammatory response but also affects the proliferation of tumor cells.

## Discussion

The immune system is responsible for the body’s immune responses and immune functions. It mainly comprises immune organs, immune cells and immune molecules. The immune system can identify and remove factors causing internal environmental fluctuations, such as foreign bodies and pathogenic microorganisms (Parkin and Cohen, 2001). In the early stage of tumorigenesis, the immune system can recognize and clear abnormally expanded cells, which is a process called immune surveillance. However, the continuous accumulation of gene mutations in tumor cells leads to increasing instability of the tumor genome and enables tumor cells to exhibit low immunogenicity and to survive (Dunn et al., 2002; Dunn et al., 2004). With the increasing malignancy of tumor cells, the growth rate of tumors is accelerated, and the tumor immune microenvironment also changes. Changes in these factors lead to the immune escape of tumor cells, and as a result, the ability of the immune system to inhibit tumors is weakened, and some tumor cells hijack the immune system to promote their proliferation (Hinshaw and Shevde, 2019). As a marker of immune cells, CD45 is used to enrich for and to sort immune cells, and it also plays an important role in a variety of cancers (Barford et al., 1994; Ozdemirli et al., 1996; Ratei et al., 1998; Rheinlander et al., 2018). However, pan-cancer research on CD45 has not been systematically conducted.

We first analyzed the expression level of CD45 at the mRNA and protein levels in a variety of cancers. We found that the expression level of CD45 in several tumors was higher than that in normal tissues. At the cellular level, CD45 was mainly expressed on immune cells, indicating the specificity of its expression, which was also the research basis for the use of CD45 as a surface marker of immune cells. The expression level of CD45 was also diverse across different kinds of immune cells, which might be related to the function of these cells in different states.

Epigenetic modifications, such as DNA methylation, histone modification, chromatin remodeling and RNA modification, can mediate regulation of gene expression in different ways, among which DNA methylation plays an important role in gene expression (Portela and Esteller, 2010; Jones, 2012). Therefore, we downloaded tumor methylation data from TCGA and analyzed the methylation level of CD45 by bioinformatics methods. We found that expression level of CD45 was the higher in TGCT and PAAD than that in UCS and PCPG. We also discovered that the correlation between the CD45 methylation level and its expression was low in TGCT and PAAD and high in UCS and PCPG. These results indicate that methylation plays an important role in CD45 expression among some tumors.

Gene expression levels can be used to predict the prognosis of cancer patients. Previous studies reported that the high expression level of CD45 is related to the poor prognosis of acute lymphoblastic leukemia (Balasubramanian et al., 2021) but the role of CD45 in prognosis across different cancers is unclear. Combining the expression level of CD45 with the clinical prognosis data from tumor patients, we found that the expression level of CD45 was significantly correlated with the prognosis of multiple tumors. UVM and LGG patients with high expression levels of CD45 had a poor prognosis, and LUAD, SKCM and HNSC patients with low expression levels of CD45 also had a poor prognosis. We used univariate analysis to analyze the relationship between CD45 expression and prognostic indicators (OS, DSS, PFI and DFI). The results showed that CD45 expression was a risk factor for these prognostic indicators in a variety of tumors.

CD45 is mainly expressed in immune compartment rather than epithelial compartment. Many studies have found that the abundance of tumor-infiltrating cells is associated with patient prognosis (Gentles et al., 2015; Wang et al., 2019; Chen et al., 2020). In addition, some studies have reported that CD45 has prognostic significance in tumor (Behm et al., 1992; Ishizawa et al., 2019; Oka et al., 2020; Balasubramanian et al., 2021). We think that combining CD45 expression with infiltration of specific immune cells may improve the accuracy of predicting patient prognosis and its potential clinical applications.

Previous studies reported that CD45 affected cell differentiation and proliferation in hematopoietic stem cells (HSCs) (Dornan et al., 2002; Hermiston et al., 2003). We performed GSEA and KEGG analysis based on the genes differentially expressed between patients with high and low expression levels of CD45 to identify potential mechanisms. The results showed that CD45 may impact tumor progression by affecting immune- and metabolic-related signaling pathways. Most tumors showed a positive correlation between CD45 and MKI67 expression suggesting that high CD45 expression is indicative of a tumor microenvironment that promotes tumor cell proliferation and cancer progression.

Immunotherapy has proved to be an important method of tumor treatment in addition to surgical treatment, radiotherapy and chemotherapy. Checkpoint inhibitors targeting PD1, PD-L1 and CTLA-4 has improved survival in many cancers (Kennedy and Salama, 2020; Nam et al., 2020). As a predictor of response to checkpoint inhibitors, TMB, TNB, MSI and MMR have demonstrated utility in some cancers (Samstein et al., 2019; Schrock et al., 2019; Lizardo et al., 2020). We observed that the expression level of CD45 was highly correlated with TMB, TNB and MSI status in COAD. It is however unclear whether this relationship is direct or indirect, given that MSI is directly associated with TMB and the latter with TNB(Schumacher and Schreiber, 2015; Goodman et al., 2019).

Although cancer immunotherapy has proved beneficial to many cancer patients, a subset of patients experiences adverse inflammatory response after immunotherapy (Crusz and Balkwill, 2015; Dougan et al., 2021). Alleviating inflammation therefore becomes an important damage control mechanism to improve long-term quality of life for these patients. Identifying targets associated with hyperinflammatory phenotype has the potential to improve patient response to cancer immunotherapy and improve their quality of life. This study has explored the use of CD45 expression to identify targets associated with inflammation that may be modulated with immunotherapy drugs in proof-of-concept studies.

By comparing tumors belonging to high and low immune index groups based on the expression of CD45, we identified the immune-related G-protein coupled receptor GPR84, a gene previously implicated in inflammation and the innate immune response (Recio et al., 2018; Marsango et al., 2020; Zhang et al., 2021). GPR84 expression was positively correlated in our analysis with expression of many proinflammatory factors found in inflamed tumors. Further studies are required to understand the mechanism of GPR84 function in the tumor microenvironment, and validate its role as a novel drug target to suppress inflammation.

In this study, we have performed a comprehensive analysis of CD45 expression using a multi-omics approach by combining data from different databases. However, this study has the following limitations. First, the source of the tumor protein data was immunohistochemical staining rather than more quantitative proteomics methods. Second, our interesting finding of GPR84 as a potential drug target came from differential expression analysis, lacking more direct evidence as to how GPR84 is linked to tumor cell proliferation. Third, our study revealed that CD45 expression may have utility in predicting prognosis of some cancers, without identifying a mechanistic basis for this observation.

## Data Availability

The original contributions presented in the study are included in the article/Supplementary Material further inquiries can be directed to the corresponding authors.
