# Supplementary material for: A multi-omic approach reveals utility of CD45 expression in prognosis and novel target discovery
Source: Front Genet. 2022 Aug 17;13:928328. doi: 10.3389/fgene.2022.928328 (PMC9428580; doi:10.3389/fgene.2022.928328)
Supplement: Supplementary file 1 [file DataSheet1.PDF]

Supplementary Material

Supplementary Figures

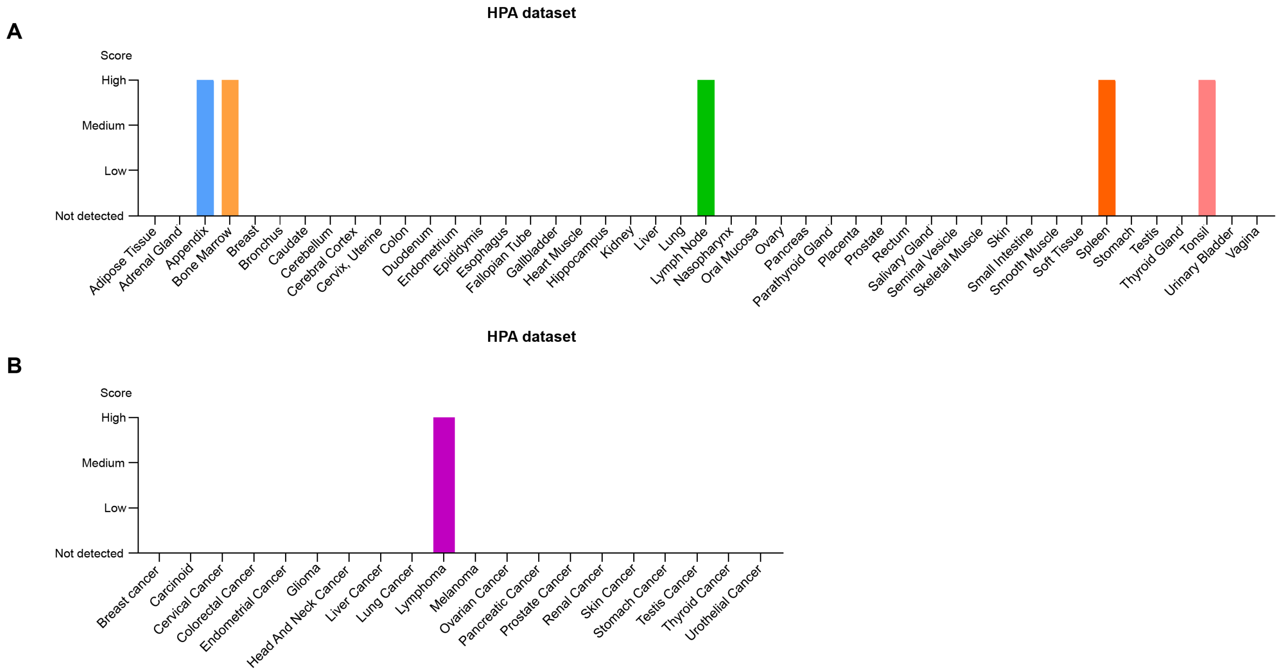

Supplementary Figure S1. The protein expression levels of CD45 in human normal tissues (**A**) and tumor tissues (**B**) is represented by quantitative data from immunohistochemical images.

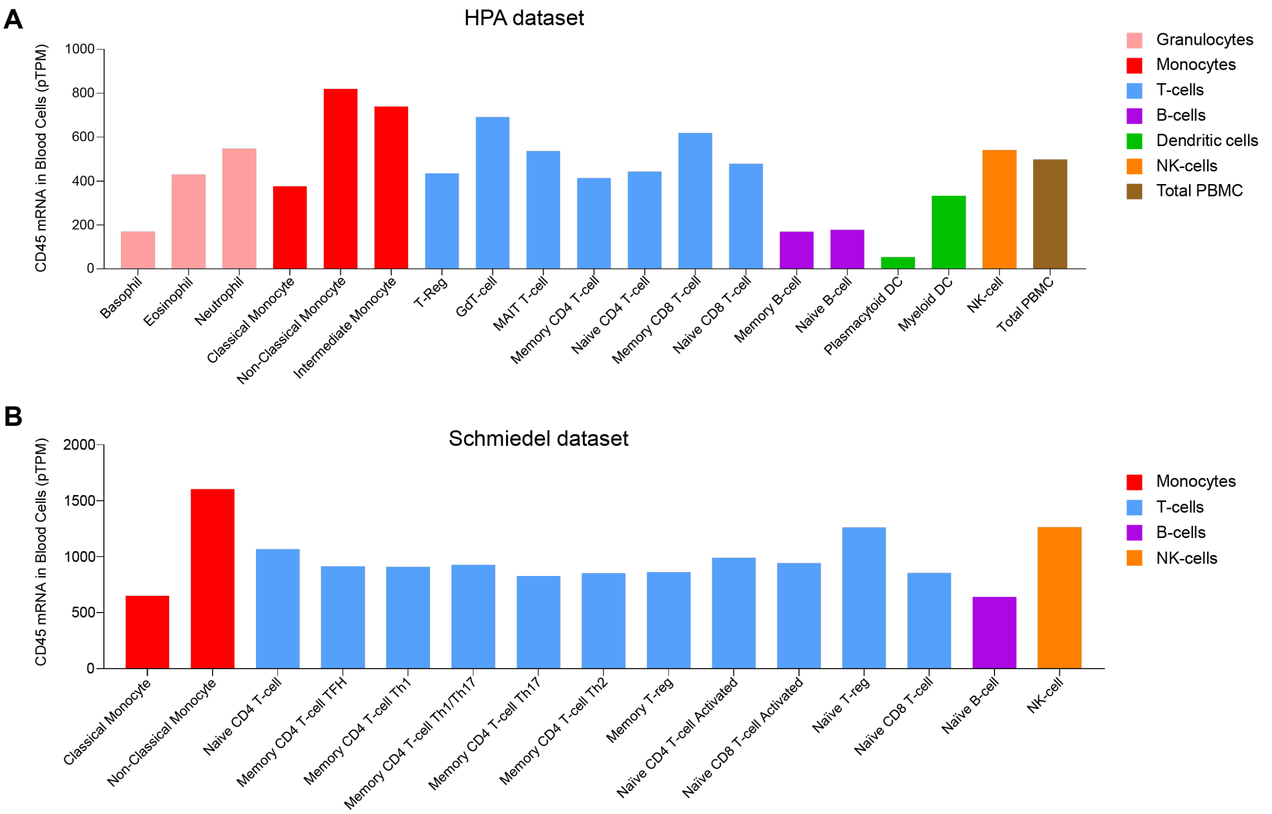

Supplementary Figure S2. Expression of CD45 in different immune cell datasets, such as the HPA (**A**) and Schmiedel dataset (**B**).

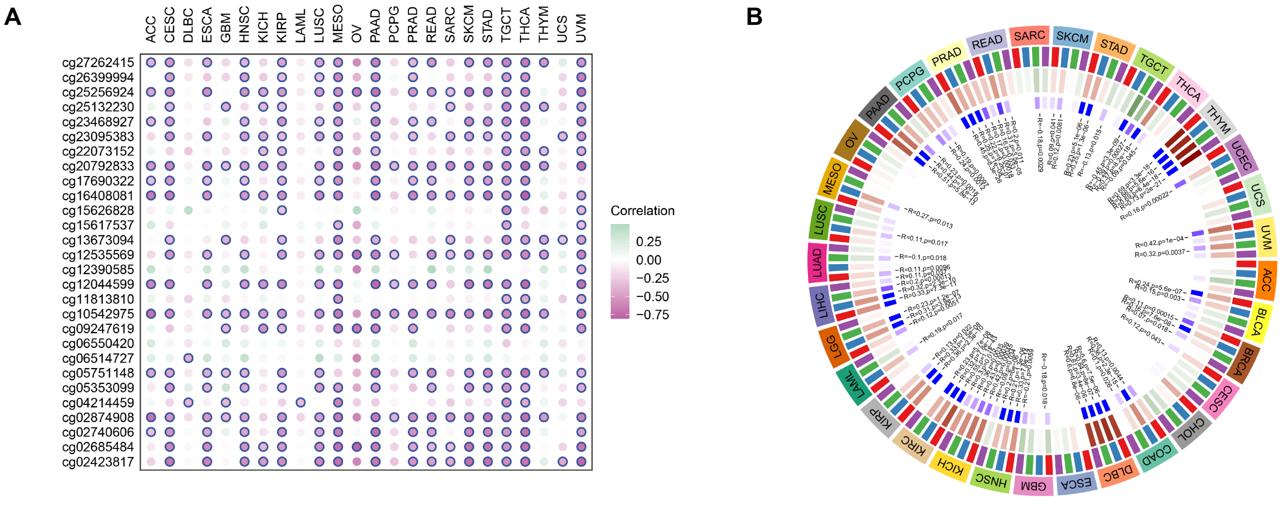

Supplementary Figure S3. The landscape of CD45 methylation across cancers. (**A**) Analysis of correlation between the methylation degree of different probes and CD45 expression in each cancer. (**B**) Analysis of correlation between CD45 expression and levels of methyltransferase-related genes in each cancer.

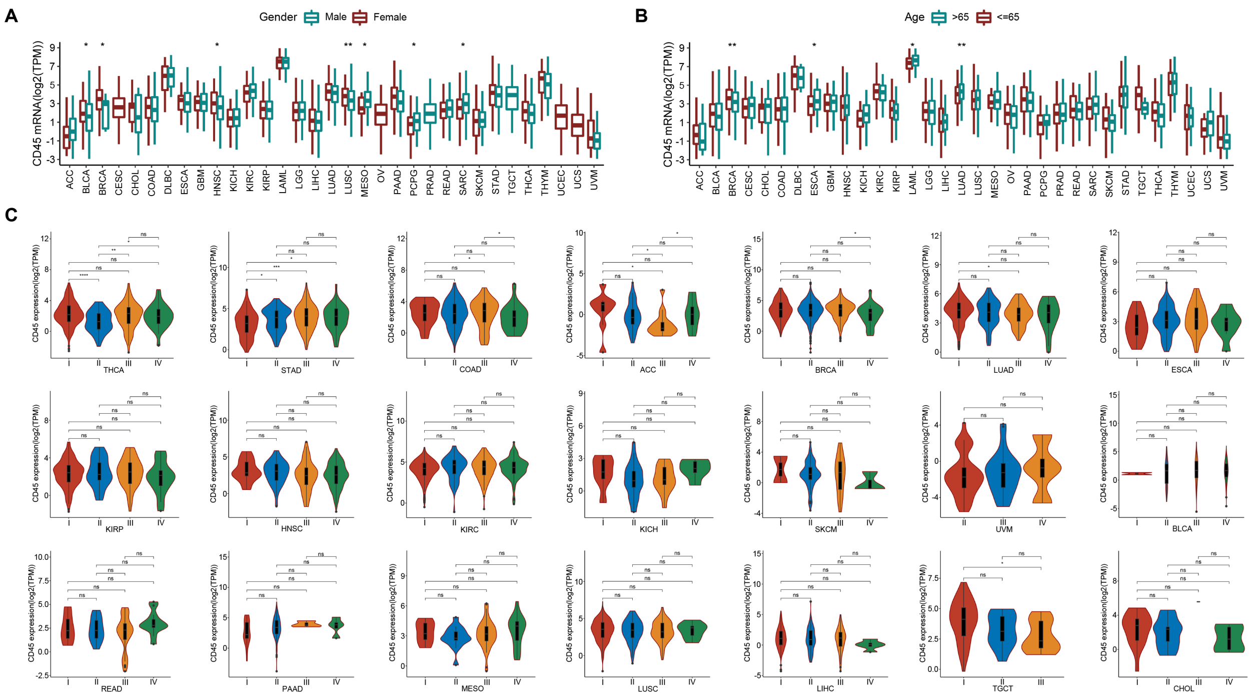

Supplementary Figure S4. Analysis of the relationship between the expression level of CD45 and clinicopathological features of tumor patients. (**A**) Pan-cancer analysis of CD45 expression across male and female patients from TCGA. (**B**) Pan-cancer analysis of CD45 expression across the high (≥65) and low (< 65) age groups of patients from TCGA. (**C**) Pan-cancer analysis of CD45 expression in tumor stages ranging from grade I to grade IV. *p < 0.05; **p < 0.01 and ***p < 0.001.-

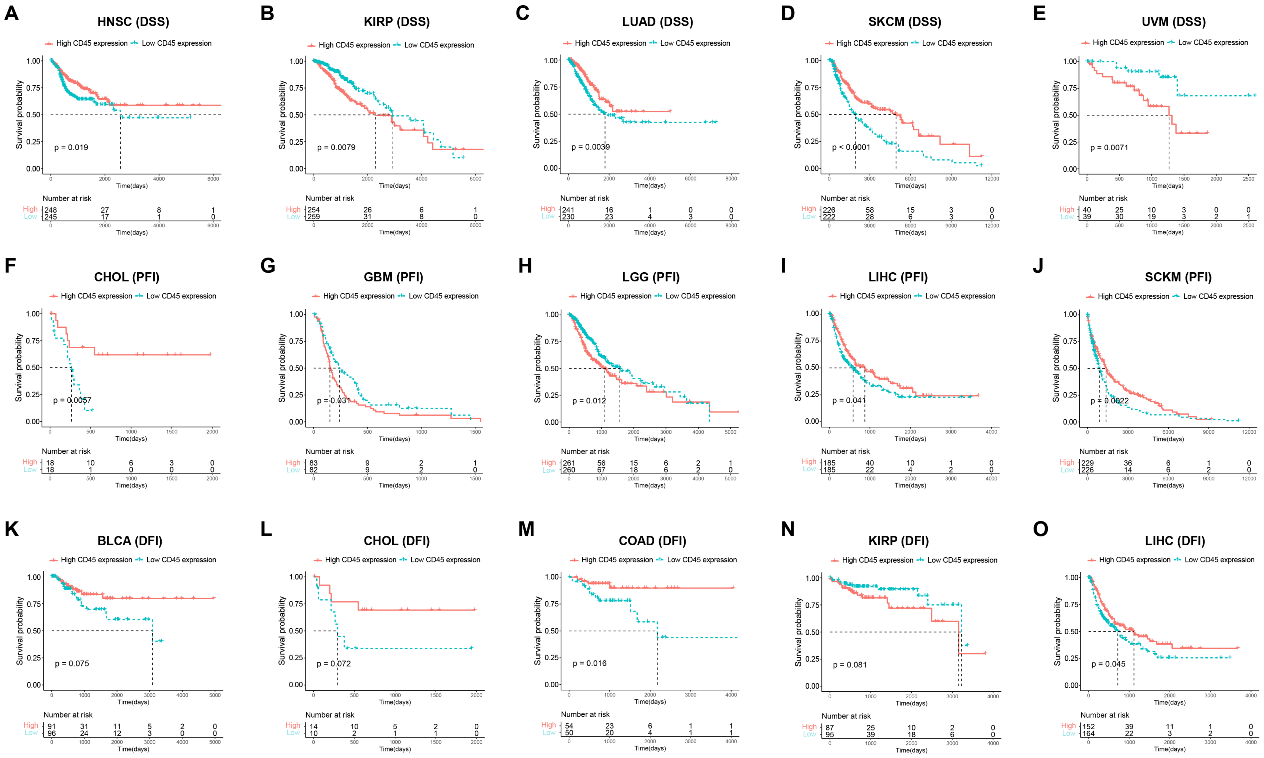

Supplementary Figure S5. Analysis of the prognostic value of CD45 across cancers. Survival curve (DSS) plotted by the Kaplan–Meier method in HNSC (**A**), KIRP (**B**), LUAD (**C**), SKCM (**D**) and UVM (**E**) in the high and low CD45 expression groups. Survival curve (PFI) plotted by the Kaplan–Meier method in CHOL (**F**), GBM (**G**), LGG (**H**), LIHC (**I**) and SCKM (**J**) in the high and low CD45 expression groups. Survival curve (DFI) plotted by the Kaplan–Meier method in BLCA (**K**), CHOL (**L**), COAD (**M**), KIRP (**N**) and LIHC (**O**) in the high and low CD45 expression groups.

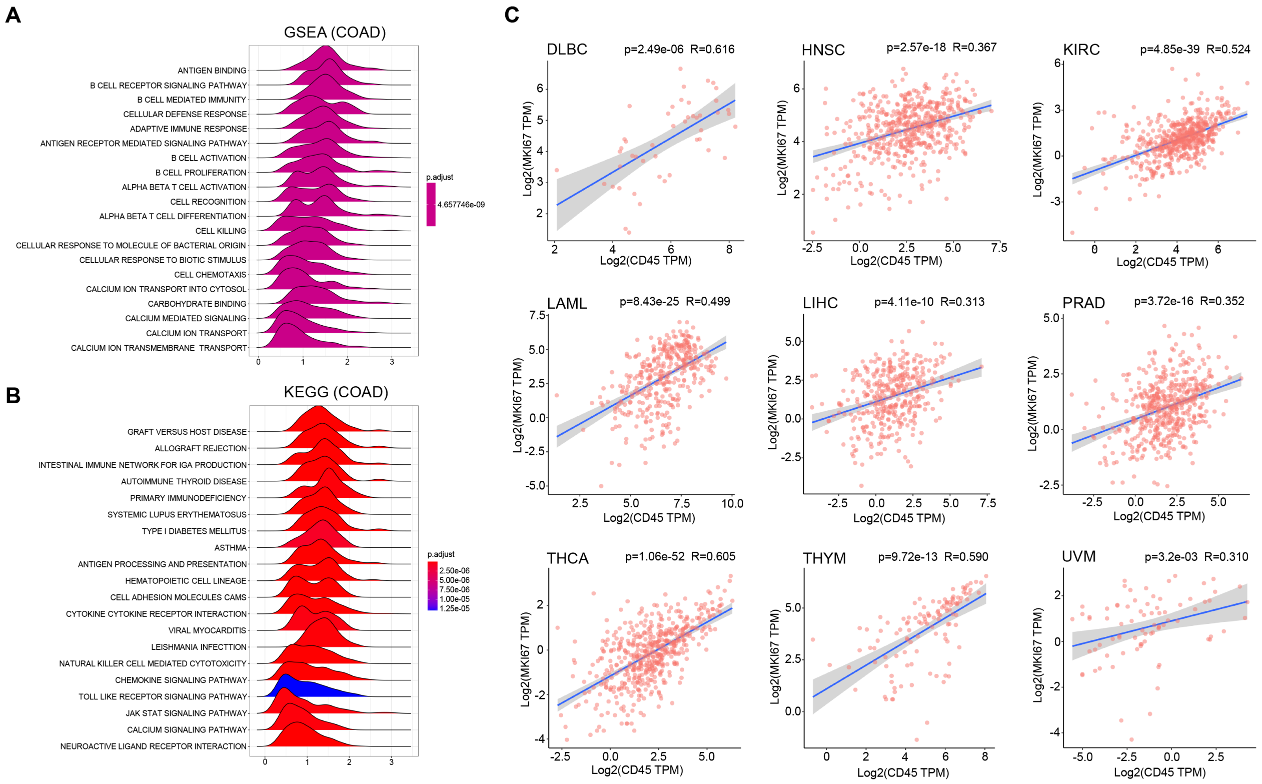

Supplementary Figure S6. Analysis of the potential mechanism of CD45 across cancers. Top 20 pathways enriched in the GO (**A**) and KEGG (**B**) analysis-based differentially expressed genes obtained between high and low CD45 patients in COAD. (**C**) Analysis of correlation between MKI67 and CD45 expression in DLBC, HNSC, KIRC, LAML, LIHC, PRAD, THCA, THYM and UVM.

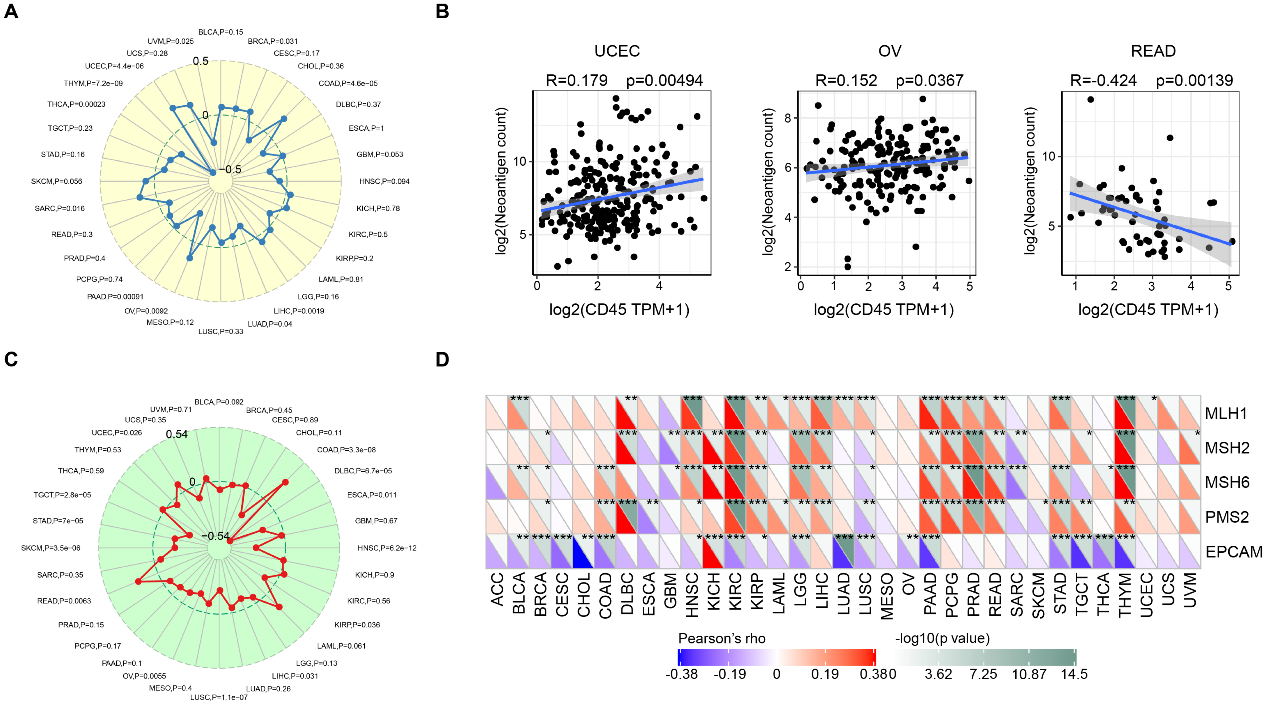

Supplementary Figure S7. CD45 correlates with TMB, TNB and MSI to predict immunotherapy effects. Analysis of correlation between CD45 expression and TMB (**A**) and MSI (**C**) across cancers. (**B**) Analysis of correlation between CD45 expression and neoantigens in UCEC, OV, and READ. (**D**) Analysis of correlation between CD45 and MMR-related genes in multiple tumors. *p < 0.05; **p < 0.01 and ***p < 0.001.

Supplementary Table

Supplementary Table S1. Abbreviation and full name of all cancers in TCGA.

Abbreviation of cancer	Full name of cancer
ACC	Adrenocortical carcinoma
BLCA	Bladder urothelial carcinoma
BRCA	Breast invasive carcinoma
CESC	Cervical squamous cell carcinoma
CHOL	Cholangiocarcinoma

COAD	Colon adenocarcinoma
DLBC	Diffuse large B cell lymphoma
ESCA	Esophageal carcinoma
GBM	Glioblastoma multiforme
HNSC	Head and neck squamous cell carcinoma
KICH	Kidney chromophobe
KIRC	Kidney renal clear cell carcinoma
KIRP	Kidney renal papillary cell carcinoma
LAML	Acute myeloid leukemia
LGG	Brain lower grade glioma
LIHC	Liver hepatocellular carcinoma
LUAD	Lung adenocarcinoma
LUSC	Lung squamous cell carcinoma
MESO	Mesothelioma
OV	Ovarian serous cystadenocarcinoma
PAAD	Pancreatic adenocarcinoma
PCPG	Pheochromocytoma and paraganglioma
PRAD	Prostate adenocarcinoma
READ	Rectum adenocarcinoma
SARC	Sarcoma
SKCM	Skin cutaneous melanoma
STAD	Stomach adenocarcinoma

TGCT	Testicular germ cell tumors
THCA	Thyroid carcinoma
THYM	Thymoma
UCEC	Uterine corpus endometrial carcinoma
UCS	Uterine carcinosarcoma
UVM	Uveal melanoma
